# Urban Transit System Microbial Communities Differ by Surface Type and Interaction with Humans and the Environment

**DOI:** 10.1128/mSystems.00018-16

**Published:** 2016-06-28

**Authors:** Tiffany Hsu, Regina Joice, Jose Vallarino, Galeb Abu-Ali, Erica M. Hartmann, Afrah Shafquat, Casey DuLong, Catherine Baranowski, Dirk Gevers, Jessica L. Green, Xochitl C. Morgan, John D. Spengler, Curtis Huttenhower

**Affiliations:** a Harvard T.H. Chan School of Public Health, Boston, Massachusetts, USA; b Broad Institute, Cambridge, Massachusetts, USA; c University of Oregon, Eugene, Oregon, USA; University of California, San Diego

**Keywords:** built environment, microbiome, subway, transit

## Abstract

Public transit systems are ideal for studying the urban microbiome and interindividual community transfer. In this study, we used 16S amplicon and shotgun metagenomic sequencing to profile microbial communities on multiple transit surfaces across train lines and stations in the Boston metropolitan transit system. The greatest determinant of microbial community structure was the transit surface type. In contrast, little variation was observed between geographically distinct train lines and stations serving different demographics. All surfaces were dominated by human skin and oral commensals such as *Propionibacterium*, *Corynebacterium*, *Staphylococcus*, and *Streptococcus*. The detected taxa not associated with humans included generalists from alphaproteobacteria, which were especially abundant on outdoor touchscreens. Shotgun metagenomics further identified viral and eukaryotic microbes, including *Propionibacterium* phage and *Malassezia globosa*. Functional profiling showed that *Propionibacterium acnes* pathways such as propionate production and porphyrin synthesis were enriched on train holding surfaces (holds), while electron transport chain components for aerobic respiration were enriched on touchscreens and seats. Lastly, the transit environment was not found to be a reservoir of antimicrobial resistance and virulence genes. Our results suggest that microbial communities on transit surfaces are maintained from a metapopulation of human skin commensals and environmental generalists, with enrichments corresponding to local interactions with the human body and environmental exposures.

**IMPORTANCE** Mass transit environments, specifically, urban subways, are distinct microbial environments with high occupant densities, diversities, and turnovers, and they are thus especially relevant to public health. Despite this, only three culture-independent subway studies have been performed, all since 2013 and all with widely differing designs and conclusions. In this study, we profiled the Boston subway system, which provides 238 million trips per year overseen by the Massachusetts Bay Transportation Authority (MBTA). This yielded the first high-precision microbial survey of a variety of surfaces, ridership environments, and microbiological functions (including tests for potential pathogenicity) in a mass transit environment. Characterizing microbial profiles for multiple transit systems will become increasingly important for biosurveillance of antibiotic resistance genes or pathogens, which can be early indicators for outbreak or sanitation events. Understanding how human contact, materials, and the environment affect microbial profiles may eventually allow us to rationally design public spaces to sustain our health in the presence of microbial reservoirs.

**Author Video**: An author video summary of this article is available.

## Abstract (precis)

Mass transit environments, specifically, urban subways, are distinct microbial environments with high occupant densities, diversities, and turnovers, and they are thus especially relevant to public health. Despite this, only three culture-independent subway studies have been performed, all since 2013 and all with widely differing designs and conclusions. In this study, we profiled the Boston subway system, which provides 238 million trips per year overseen by the Massachusetts Bay Transportation Authority (MBTA). This yielded the first high-precision microbial survey of a variety of surfaces, ridership environments, and microbiological functions (including tests for potential pathogenicity) in a mass transit environment. Characterizing microbial profiles for multiple transit systems will become increasingly important for biosurveillance of antibiotic resistance genes or pathogens, which can be early indicators for outbreak or sanitation events. Understanding how human contact, materials, and the environment affect microbial profiles may eventually allow us to rationally design public spaces to sustain our health in the presence of microbial reservoirs.

## INTRODUCTION

Mass transit systems host large volumes of passengers and facilitate a constant stream of human/human and human/built environment microbial transmission. The largest urban mass transit system in the United States (that in New York) facilitates an average of 11 million trips per weekday. The next four largest systems (those in Washington, DC; Chicago; Boston; and San Francisco) transport just over 1 million passengers per weekday (1), and yet little is known about the mass transit system microbial reservoir. Understanding the associated dynamics of microbial transmission between humans and the built environment and those of microbial occupation and persistence on different surfaces can inform decisions regarding public health and safety.

Microbial DNA sequencing-based studies have revealed that microbial communities of the built environment are greatly influenced by their human occupants. Communities within homes showed high similarity to those of their inhabitants (2), and specific surfaces frequently contacted by human skin, such as keyboards or mobile phones, had microbial communities that reflected those of skin (3, 4). In restrooms and classrooms, variation in microbial community composition across surface types was associated with variations in human contact with those surfaces: desks contained human skin and oral microbes, while chairs contained intestinally and urogenitally derived microbes (5, 6). However, a limitation of most built environment microbiome research is that human contact, surface type, and material composition are frequently confounded. For example, in the classroom study described above, different forms of human contact were associated with distinct microbial community profiles; however, the desks and chairs were also constructed from different materials.

Previously observed subway microbial communities comprised microbes from both humans and the environment. Air samples from within the New York and Hong Kong subway systems included microbes originating from soil and environmental water in addition to human skin (7, 8). A recent metagenomic study of New York subway stations (9) has been widely criticized (10) and left unanswered many questions regarding detailed analysis of the transit microbiome, but it has provided an initial reference data set for further analysis of subway microbiome diversity. In addition, while that study collected information regarding surface types, it did not standardize their characterization or, as a result, investigate surface-specific enrichments for microbial taxa. Understanding the separate influences of human contact, surface type, and surface material would help identify mechanisms through which microbial communities form and persist on surfaces within built environments.

In the present report, we provide the first comprehensive metagenomic profile of microbial communities across multiple surface types and materials in a high-volume public transportation system. Samples were collected from seats, seat backs, walls, vertical and horizontal poles, and hanging grips inside train cars in three subway lines, as well as from touchscreens and walls of ticketing machines inside five subway stations. Using a combination of 16S amplicon and shotgun metagenomic sequencing, we characterized the microbial community composition, functional capacity, and pathogenic potential of the Boston mass transit system. In agreement with previous studies, we observed combinations of human-, soil-, and air-derived microbial communities across the system. Taxonomic differences were most strongly associated with surface type, in contrast to geographic, train line, and material differences, in a multivariate analysis. The distribution of metabolic functions was dominated by *Propionibacterium acnes* bacteria, which made up a majority of the community. Minimal antibiotic resistance genes and virulence factors were detected across transit system surfaces. In addition to identifying the most important factors determining microbial colonization, our results may serve as a baseline description of microbes on public transportation surfaces, which will be relevant for future design of healthy transit environments.

## RESULTS

### Sampling microbial communities in the Boston transit system.

**FIG 1:**
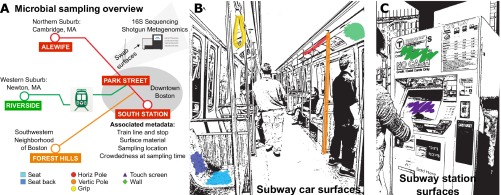
Collection of samples from MBTA trains and stations. (A) Microbial community samples were collected from the Massachusetts Bay transit system in the metropolitan area of Boston, MA. Train samples were collected from 6 train car surfaces across 3 locations along 3 train routes; station samples were collected from 5 stations. Horiz, horizontal; Vertic, vertical. (B and C) Diagram of the surfaces sampled within train cars (B) and stations (C). Sampled surfaces specifically included seats and seat backs, horizontal and vertical poles, hanging grips, and walls within train cars, as well as the screens and walls of touchscreen machines within stations.

We collected samples (*n* = 73) from train cars and stations in the Boston transit system. This system is maintained by the Massachusetts Bay Transportation Authority (MBTA), which operates bus, subway, commuter rail, and ferry routes in the greater Boston area. Our study focused on the subway system, which consists of five lines (red, orange, blue, green, and silver) that extend from downtown Boston into the surrounding suburbs (Fig. 1A). Train car samples were collected from the red, orange, and green lines and comprised 6 surface types, including grips, horizontal and vertical poles, seats, seat backs, and walls (Fig. 1B). Station samples were collected from the touchscreens and the sides of fare ticketing machines (Fig. 1C). Biomass yields were highest for hanging grips (141.83 ± 92.68 ng/µl), followed by seats (128.1429 ± 49.955 ng/µl) and touchscreens (120.47 ± 73.68 ng/µl), though these differences were not statistically significant (see Fig. S1A in the supplemental material).

10.1128/mSystems.00018-16.1 Figure S1  Biomass and alpha diversity for train and station samples. (A) Biomass from samples collected across the subway system. Each data point represents a pooled sampling strategy in which two or three swabs from the same site were pooled and jointly extracted. DNA yield is plotted in nanograms per milliliter. (B) Alpha diversity by surface type, as measured by the inverse Simpson diversity index. In both panel A and panel B, colors represent the line of the train from which sample was derived (red, orange, or green indicates the line of the train or station; black indicates samples collected from within a downtown station). Download Figure S1, TIF file, 0.2 MB. Copyright © 2016 Hsu et al. 2016 Hsu et al. This content is distributed under the terms of the Creative Commons Attribution 4.0 International license.

For each sample, we collected metadata describing the built environment type, surface type, and material composition as well as the collection date (see Table S1 in the supplemental material). For train car samples, we also recorded metadata describing the train line, within-train location, and location along the subway route at the time of sample collection (nearest subway stop). For station samples, we recorded the station and the ticketing machine location and which side of the touchscreen was swabbed. 16S rRNA gene amplicon sequence data were generated from most samples (*n* = 72), and those in a subset (*n* = 24) were subjected to shotgun metagenomic sequencing.

10.1128/mSystems.00018-16.5 Table S1  Sample collection and metadata. Data include metadata for all collected samples that were sequenced via 16S amplicon or shotgun sequencing. Abbreviations are defined at the bottom. Download Table S1, XLSX file, 0.02 MB. Copyright © 2016 Hsu et al. 2016 Hsu et al. This content is distributed under the terms of the Creative Commons Attribution 4.0 International license.

### Microbial communities are specific to surface types and the immediate environment.

**FIG 2:**
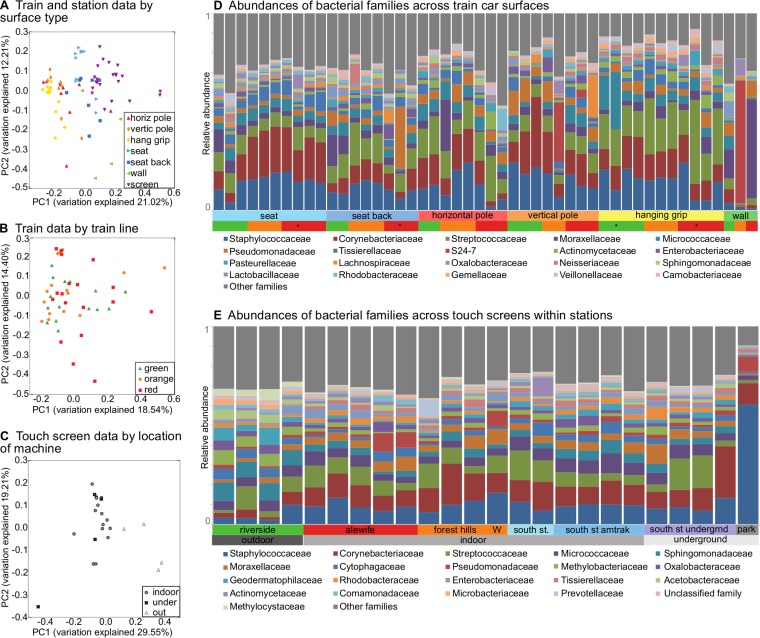
Taxonomic composition of subway microbial communities. All ordinations are from principal coordinate analyses using Bray-Curtis distances among filtered OTUs (see Materials and Methods), colored by metadata. (A) Subway data by surface. PC1, principal coordinate 1; PC2, principal coordinate 2. (B) Train car data by train line. (C) Touchscreen data by location of machine. (D) Relative abundances of bacterial families across samples from train cars (see Table S2 in the supplemental material for complete data). (E) Relative abundances of bacterial families within stations (complete data determined as described above). Stars indicate that the sample was collected on a separate day during the same month as the remaining samples. For station samples, “W” indicates a sample from a ticketing machine wall; all other samples were from the ticketing machine touchscreens.

The surface type from which microbes were collected proved to be the major determinant of community diversity and structure. The alpha diversity of touchscreen samples was significantly higher than that of all other surface types (*P* < 0.0001 for comparison of 7 surfaces by analysis of variance [ANOVA] with Bonferroni correction; see Fig. S1B in the supplemental material) and did not correlate with biomass (Spearman’s rho = 0.0057; see Fig. S1A). The largest axes of beta diversity separated train holding surfaces (holds) (horizontal and vertical poles, hanging grips), chairs (seat and seat backs), touchscreens, and walls (Fig. 2A). The train line remained only a minor driver of community structure (Fig. 2B) and did not dictate overall community composition for either holds (see Fig. S2A) or seats, once the material of the latter was taken into account (see Fig. S2B and C). In particular, the seats on the green line were upholstered with vinyl, while the seats on the orange and red lines were upholstered with polyester.

10.1128/mSystems.00018-16.2 Figure S2  Ordination of surface data subsets. (A) Train holding surfaces by train line. (B) Train chair surfaces by train line. (C) Train chairs by material. (D) Touchscreen surfaces by date. All ordination data represent results of principal coordinate analyses performed using Bray-Curtis distances, colored by metadata category and calculated using filtered OTU relative abundance table subsets of the relevant samples. Download Figure S2, TIF file, 0.8 MB. Copyright © 2016 Hsu et al. 2016 Hsu et al. This content is distributed under the terms of the Creative Commons Attribution 4.0 International license.

10.1128/mSystems.00018-16.6 Table S2  16S and shotgun OTU tables along with taxa present across sequencing plate. The first tab presents the 16S OTU counts after quality control, stitching, and length filtering, after removal of chloroplasts, mitochondria, and archaea, and after filtering for at least 0.1% in 1 sample. The second tab presents the unfiltered MetaPhlAn OTU table with percentages (100 = 100%). Note that additional filtering was performed before LEfSe and MaAsLin runs for both 16S (at least 0.1% in 7 samples) and shotgun (at least 0.1% in 2 samples) analyses. The third tab presents results of our analyses performed to identify contaminant taxa. For a negative control, we examined all samples present on a sequencing plate containing a subset of MBTA samples, which included samples from touchscreens (*n* = 21) and trains (*n* = 6), 30 saliva cultures, 13 skin samples, and 2 macaque tissue samples. Listed taxa were present in 80% of samples with at least 0.00001 abundance and are shown with their average abundance across all samples. This provides a quality control test for potential contaminant taxa, none of which were abundant or significant during our MBTA analyses. Download Table S2, XLSX file, 0.7 MB. Copyright © 2016 Hsu et al. 2016 Hsu et al. This content is distributed under the terms of the Creative Commons Attribution 4.0 International license.

The location of ticketing machines (e.g., outdoor, indoor, or underground) was a primary source of variation between microbial communities on touchscreens (Fig. 1C). Univariate analyses performed using linear discriminant analysis effect size (LEfSe) (11) revealed that indoor touchscreens were characterized by the presence of species of the *Acinetobacter* genus, while underground touchscreens had increased levels of species of the *Corynebacterium* genus and *Tissierellaceae* family, specifically, species of the *Finegoldia* genus and *Anaerococcus* genus. Those with outdoor exposures were enriched for species of the *Alphaproteobacteria* class, including members of the family *Acetobacteraceae* and genera *Methylobacterium*, *Sphingomonas*, and *Blastococcus* (see Table S3 in the supplemental material). These results imply that surface type is a major driver of community composition on transit surfaces and that indoor exposure versus outdoor exposure detectably influences the resident microbial composition of touchscreen surfaces.

10.1128/mSystems.00018-16.7 Table S3  LEfSe and MaAsLin analysis for 16S sequencing. The first tab contains LEfSe results of searches for differentially abundant taxa between touchscreen locations (outdoor [out], underground [under], and indoor near an exit facing an outside environment [inout]). Significant results represent both logarithmic LDA scores and *P* values. The second tab presents results of MaAsLin analyses run with four covariates, including surface category, surface type, surface material, and surface location. Only organisms with *q* values of >0.25 are reported. Download Table S3, XLSX file, 0.1 MB. Copyright © 2016 Hsu et al. 2016 Hsu et al. This content is distributed under the terms of the Creative Commons Attribution 4.0 International license.

### Subway microbial communities are largely derived from human skin and oral commensal microbes.

**FIG 3:**
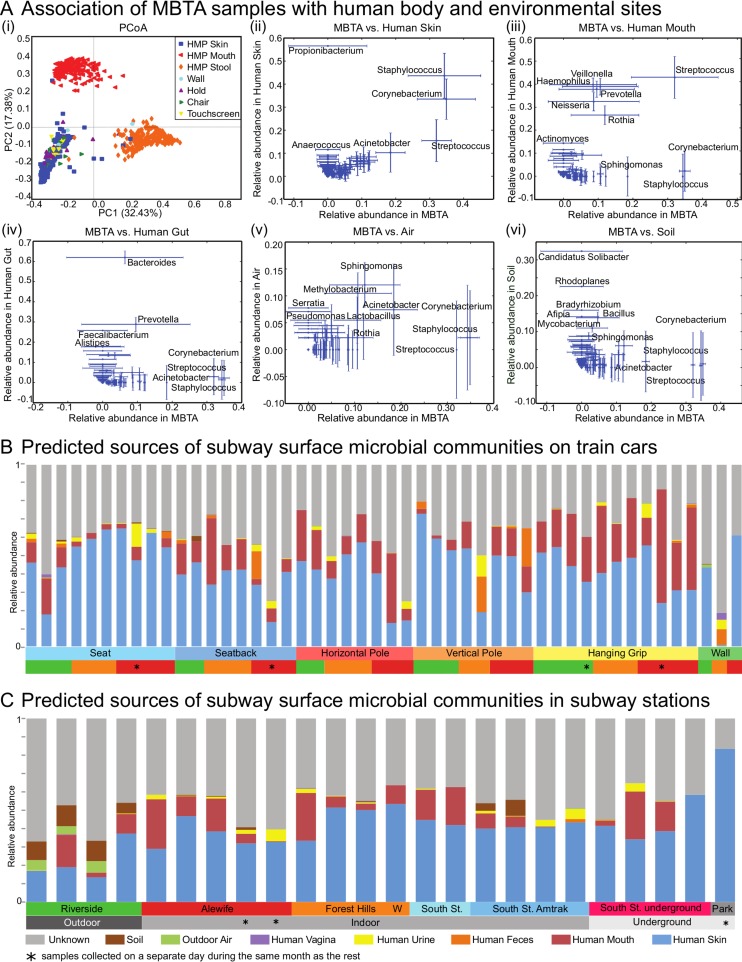
Putative MBTA microbial community sources. (Ai) Ordination of subway surface data jointly with human skin (anterior nares), oral (mixed sites from within oral cavity), and gut (stool) microbiome data from the Human Microbiome Project (HMP) (12). Principal coordinate analysis was performed with weighted UniFrac distance and calculated using OTU relative abundances. (Aii to vi) Correlations between subway samples and human body sites (19), including skin (ii), oral (iii), and gut (iv) samples, as well as environmental sites, i.e., air (20) (v) and soil (21) (vi) samples. The *x* and *y* axes represent mean relative abundance levels across each data set with standard error bars. For each plot, subway samples (MBTA) are represented on the *x* axis and potential source community samples are represented on the *y* axis. (B and C) Microbial SourceTracker (22) was used to identify possible human and environmental sources of subway (B) train and (C) station communities. The relative estimated contribution of each source is plotted per subway sample.

Subway microbial clades were generally those found in typical human skin communities (12, 13) (Fig. 3Ai) and were dominated by members of the phyla *Firmicutes*, *Proteobacteria*, and *Actinobacteria*, each of which comprised over 20% of the microbial community, based on 16S data. The members of *Bacteroidetes* were much less abundant, with an average community abundance of 6% (see Table S2 in the supplemental material). The families of species with the highest mean relative abundances were *Staphylococcaceae* and *Corynebacteriaceae* (Fig. 2D and E), which are also typical of skin commensals. The presence of *Propionibacterium* spp. was not observed due to known primer bias (14) but was confirmed later with shotgun metagenomics. The next-most-abundant taxa were *Micrococcaceae*, which included genus *Micrococcus* (found in hair and skin) and genus *Rothia* (found in the oral cavity [12, 15]), and *Streptococcaceae* (found in the oral cavity) and *Pseudomonadaceae*. We also observed low proportions of gut and oral commensals such as *Lachnospiraceae*, *Veillonella*, and *Prevotella*.

Highly abundant taxa not associated with humans encompassed the order *Burkholderiales* (3.25%) and the class *Alphaproteobacteria* (9.15%), which contains genera *Sphingomonas* (1.48%) and *Methylobacterium* (1.14%) and families *Rhodobacteraceae* (1.48%) and *Methylocystaceae* (0.447%). These alphaproteobacteria are widespread environmental bacteria with flexible metabolic regimes; in particular, sphingomonads, including the genera *Sphingomonas* and *Sphingobium*, are found in soils and sediments and are well studied for their ability to degrade polyaromatic hydrocarbons (16). Species of *Methylobacterium*, in particular, *M. extorquens*, comprise a genus of plant- and soil-associated facultative methylotrophs; these bacteria are highly prevalent on the surfaces of plants, and their diverse metabolic capabilities make them likely to survive in other environments (17). *Enhydrobacter aerosaccus*, which is currently classified as belonging to *Moraxellaceae* but may more aptly be classified as an alphaproteobacterium (18), was also prevalent in the subway samples.

To determine the microbial clades driving these patterns, we correlated the abundances of subway microbial genera with their abundances in three human body sites (19) as well as in air and soil (20, 21) (Fig. 3Aii to vi). As expected, members of the human skin genera *Staphylococcus* and *Corynebacterium* (Fig. 3Aii), the human oral cavity taxon *Streptococcus*, and the human gut-resident genera *Bacteroides* and *Prevotella* are abundant on both the subway and their respective body sites (Fig. 3Aii to iv). In addition to human-associated taxa, members of several genera previously observed in indoor air (20), *Sphingomonas*, *Methylobacterium*, *Acinetobacter*, *Streptococcus*, *Staphylococcus*, and *Corynebacterium*, were also abundant on subway surfaces (Fig. 3Av). In contrast, typical soil genera were rare on subway surfaces (Fig. 3Avi). Microbial SourceTracker (22) confirmed these origins based on overall community composition compared to a variety of reference environments (23) (Fig. 3B and C). Only a subset of touchscreen samples included a substantial proportion of environmental microbes (e.g., air and soil), most notably from the Riverside aboveground outdoor ticketing station (Fig. 3C).

### *Propionibacterium* phages and the yeast *Malassezia globosa* dominate the nonbacterial microbial community.

**FIG 4:**
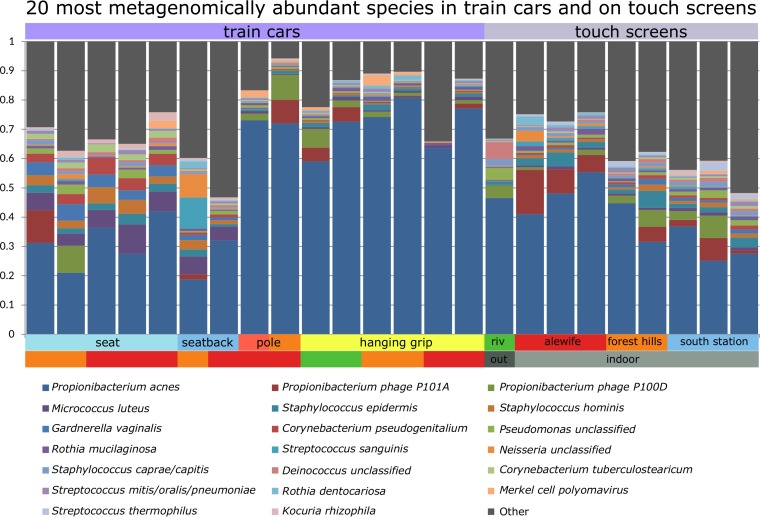
Transdomain taxonomic profiles from subway shotgun metagenomes. Data represent the relative abundances of the 20 microbial species with the highest mean across 24 metagenomes from train cars and stations. Among colored metadata annotations, train line data (green, orange, or red) are indicated for car surface samples and location data (indoor or outdoor) for touchscreens. *P. acnes* is not amplified by the 16S primers used in this study but is readily detectable by shotgun sequencing, as are nonbacteria such as *Propionibacterium* phage.

Shotgun metagenomic sequencing, which allowed us to profile viral and eukaryotic microbes that cannot be identified by 16S sequencing as well as bacterial taxa that are poorly amplified by the 16S V4 region primers (14), was performed for 24 mass transit samples, including 15 train car samples and 9 station samples. In agreement with previous studies of skin ribotypes (13, 24), the most abundant species across all samples was the facultative anaerobe *P. acnes* (mean abundance, 47%; maximum abundance, 81%); its average abundance was 29.8% for chairs, 71.6% for grips and poles, and 43.4% for touchscreen surfaces (Fig. 4). Other metagenomically assessed bacterial abundances agreed with 16S data, including high levels of members of the family *Micrococcaceae* (mean, 5.3%), *Staphylococcaceae* (mean, 5.28%), *Corynebacteriaceae* (mean, 4.95%), and *Streptococcaceae* (mean, 3.73%), along with taxa not associated with humans, including the soil taxa *Geodermatophilaceae* (mean, 1.22%) and *Acinetobacter* (mean, 0.70%) (see Table S2 in the supplemental material)*.*

Eleven nonbacterial species were present at an abundance of ≥0.1% in at least two samples. The most abundant and prevalent viruses included *Propionibacterium* bacteriophages and the oncovirus Merkel cell polyomavirus (which causes a common respiratory infection [14]). The relative abundances of *Propionibacterium* bacteriophages P100D and P101A showed abundance patterns similar to those seen with *P. acnes*, with a lower average abundance on chairs (3.2%) and higher abundances on holds (5.4%) and touchscreens (7.9%), suggesting that phage/host relationships are detectable directly from metagenomics. The remaining viruses were found sporadically (in only 2 samples) or had mean relative abundances of less than 0.0006% (see Table S2 in the supplemental material). Many of these viruses were phage that corresponded to abundant bacterial species, including *Pseudomonas* phage, *Lactobacillus* phage, *Lactococcus* phage, *Staphylococcus* phage 3A, *Staphylococcus* phage 80 alpha, and *Staphylococcus* phage phi2958PVL.

The yeast *Malassezia globosa* (25) also occurred with abundance patterns similar to those seen with *P. acnes*, with a lower abundance on chairs (0.03%) and higher abundances on holds (0.25%) and touchscreens (0.1%). Both *M. globosa* and *P. acnes* show niche-specific adaptation to metabolism of lipid-rich sebum (25, 26) and are commonly found on sebaceous skin sites, which comprise the chest, back, and face (13). This may indicate that sebaceous skin taxa more easily transfer or adhere to surfaces of built environments.

### All surface types were dominated by skin microbes, with smaller proportions of oral, gut, and environmental taxa across seats and touchscreens.

**FIG 5:**
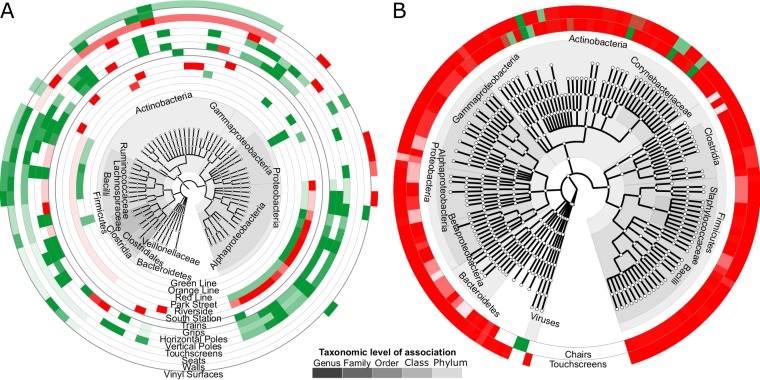
Enrichment of microbial taxa with respect to metadata using multivariate analyses. Each ring represents significant associations of one metadatum with microbial clades as determined using MaAsLin (27) (FDR *q* < 0.25). (A) 16S data. For location, surface category, surface type, and surface material (inner rings to outer rings), the direction of association between taxa and metadata relative to Alewife, touchscreens, seat backs, and polyester, respectively, is indicated in red (positive) or green (negative). (B) Shotgun metagenomic data. Only a simplified surface type was represented by a number of samples sufficient for analysis. Horizontal poles, vertical poles, and grips were grouped into holding surfaces (“holds”), and seats and seat backs were grouped into “chairs.” The direction of association is again indicated by color. Only taxa with at least one association are shown in each cladogram.

To identify differentially abundant taxa across metadata categories, we performed a multivariate analysis using MaAsLin (27), which controls for multiple covariates using a generalized linear model (see Table S3 and S4 in the supplemental material). For 16S data, we accounted for built environment type, surface type, material composition, and sample location. For human-associated taxa, seats were particularly enriched in the skin taxon *Corynebacterium* and vaginal taxon *Gardnerella*, though all contacted surface types had higher relative abundances of *Corynebacterium* spp. than train walls did (Fig. 5A). The skin taxon *Staphylococcus* was also enriched across all surface types except for touchscreens and train walls, and the presence of *Corynebacterium* spp. was negatively associated with vinyl seats relative to polyester seats. Grips were enriched for oral taxa such as *Rothia* and *Veillonella*. For taxa not associated with humans, all grips and vertical poles were depleted in species of class *Alphaproteobacteria*, as contrasted to their enrichment on outdoor surfaces at the Riverside Station (western suburb). These clades included *Methylobacteriaceae* (grips and vertical poles) and *Methylocystaceae* (all holds), as well as family *Sphingomonadaceae* (grips and vertical poles) and genus *Amaricoccus* (all holds). Because many of these organisms are likely associated with soil particles, it is reasonable that they should be less abundant on surfaces where soil is unlikely to settle.

10.1128/mSystems.00018-16.8 Table S4  MaAsLin analysis for shotgun data. MaAsLin analysis was performed to identify differentially abundant taxa (first and second tabs) and KOs (third and fourth tabs) with respect to surface type. For both analyses, surface type data were split into chairs (seat backs and seats), holds (horizontal/vertical poles, grips), and touchscreens. To identify differentially abundant taxa, we performed MaAsLin analyses with full taxonomies at all levels (first tab) as well as with species only (second tab). All results are reported; we considered results from organisms with *q* values of <0.25 to be significant. For identifying differentially abundant KOs, we performed MaAsLin analyses of KO abundances calculated both using all shotgun reads (third tab) and after *P. acnes*-associated reads were removed (fourth tab). Only significant results are reported; they represent KOs with *q* values of <0.05. Download Table S4, XLSX file, 0.2 MB. Copyright © 2016 Hsu et al. 2016 Hsu et al. This content is distributed under the terms of the Creative Commons Attribution 4.0 International license.

For shotgun data, we again used MaAsLin (27) to identify associations between microbial taxa and a single covariate, surface type (Fig. 5B; see also Table S4 in the supplemental material). Due to the small number of samples, surface type metadata were grouped into the categories of chairs (seat and seat backs), holds (hanging grips, horizontal and vertical poles), and touchscreens. For human-associated taxa, chairs and touchscreens were enriched in multiple species of *Corynebacterium* (including *C. aurimucosum*, *genitalium*, *jeikeium*, *massiliense*, *pseudogenitalium*, *tuberculostearicum*, and *urealyticum*) and *Staphylococcus* (*S. caprae capitis*, *epidermis*, *haemolyticus*, *hominis*, and *pettenkoferi*); vaginal taxa *Gardnerella vaginalis* and *Lactobacillus* (*L. crispatus* and *L. iners*); and gut taxa *Ruminococcus bromii*, *Faecalibacterium prausnitzii*, and *Eubacterium rectale*. Touchscreens were particularly enriched in oral species from genera such as *Streptococcus* (*S. cristatus*, *gordonii*, *infantis*, *mitis*/*oralis*/*pneumoniae*, *parasanguinis*, *sanguinis*, *thermophiles*, *tigurinus*), *Prevotella* (*P. copri*, *melaninogenica*), and *Rothia aeria* (also enriched on holds). For taxa not associated with humans, we saw patterns similar to those seen with the 16S data. Touchscreens were enriched in families *Methylobacteriaceae* and *Rhodobacteraceae*, as well as orders *Burkholderiales* and *Sphingomonadales* (also enriched on chairs). Many of these taxa not associated with humans that we identified on surfaces are hardy generalists that survive under harsh conditions (28).

Most *Corynebacterium* species enriched on both chairs and touchscreens had higher (but not statistically significant) abundances on chairs, with the exception of *C. kroppenstedtii* and *C. matruchotii*. The lack of oral species on holds may have been due to the newfound detection of *P. acnes*, which was enriched on holds and might affect the relative abundances of rarer taxa. Generally, skin taxa dominated all surfaces, with *P. acnes* enriched on holds and *Corynebacterium* and *Staphylococcus* on chairs and touchscreens. Oral taxa were present on both holds and touchscreens. Taxa not associated with humans remain enriched on touchscreens, which present more-exposed surface areas not enclosed within trains.

### Metagenomes reflect dominance of *P. acnes* across subway surfaces.

Functional genomic profiling using HUMAnN2 quantified 3,975,869 UniRef50 (29) protein families, which were collapsed into 12,074 KEGG Orthology (KO) (30) families. For hypothesis testing, we focused on 604 KOs with mean abundances greater than the overall median abundance and variance across samples in the 90th percentile. MaAsLin identified 590 KOs significantly associated with surface type (*q* = <0.05): 360 enriched on holds, 204 depleted on holds, 12 enriched on chairs, 4 depleted on chairs, 5 enriched on touchscreens, and 4 depleted on touchscreens (relative to all other surface types) (see Table S4 in the supplemental material).

**FIG 6:**
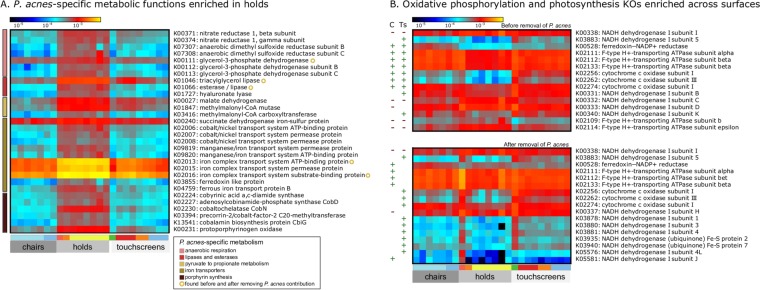
Enrichment of members of KEGG Orthology (KOs) families across MBTA surfaces before and after *P. acnes* removal. For all heat maps, rows represent significantly enriched KOs detected through linear regression performed with MaAsLin, columns represent samples, and cells are colored according to the number of sum-normalized reads per kilobase (RPKs) on a log scale. Further metadata are shown as colored bars below the heat maps. The first colored bar explains the collapsed surface types (second bar). The “chairs” category includes seats (light blue) and seat backs (dark blue); the “holds” category includes horizontal poles (red), vertical poles (orange), and grips (yellow); and the “touchscreens” category includes data from Riverside (green), Alewife (red), Forest Hills (orange), and South Station (light blue). KOs annotated with yellow circles correspond to those found before and after *P. acnes* removal. (A) Selected KOs enriched on holds only are specific to and colored according to *P. acnes* metabolic function. (B) Selected KOs specific to oxidative phosphorylation and photosynthesis are shown before (above) and after (below) *P. acnes* removal. Directions of association between KO abundances and surface types, relative to holds, are shown as a green plus sign (“+”) (positive) or a red minus sign (“−”) (negative) to the left of the heat map. Columns are colored by metadata as described for Fig. 2.

Many of the KOs enriched on holds were genes found in the *P. acnes* genome (31). These included systems for anaerobic respiration, lipases and esterases for degrading lipids within sebaceous sites, and hyaluronate lyase for digesting the extracellular matrix of skin and fermentation of pyruvate to propionate (Fig. 6A). Production of propionate is catalyzed by methylmalonyl-coenzyme A (methylmalonyl-CoA) carboxyltransferase, which is enriched on the holds. Porphyrin synthesis is a major function of several species of *Propionibacterium* (32), contributing to a range of physiological activities (e.g., potential keratinocyte damage from free radical release [31, 33]) and industrial uses (e.g., synthesis of vitamin B12 [34]). Here, the pathway was represented by several genes from the *hem* and *cbi*/*cob* gene clusters (34, 35). To verify that the KOs detected as described above were indeed specific to *P. acnes*, we removed its contributions to the overall abundance of each UniRef50 family, renormalized, and again identified KOs enriched on different surface types (see Materials and Methods). With a few exceptions, including iron transport (Fig. 6A; see also Table S4 in the supplemental material), KOs specific to *P. acnes* metabolism were no longer enriched on holds.

Many KOs associated with oxidative phosphorylation and photosynthesis were enriched on chairs and touchscreens relative to holds before removal of *P. acnes*. These included NADH dehydrogenase I subunits (EC 1.6.5.3), ferredoxin-NADP^+^ reductase (involved in photosystem I; EC 1.18.1.2), ATPase subunits (EC 3.6.3.14), and cytochrome *c* oxidases (EC 1.9.3.1). After depletion of *P. acnes*-derived processes, ferredoxin-NADP^+^ reductase and F-type H^+^-transporting ATPase subunits were enriched only on chairs, while cytochrome *c* oxidase subunits and NADH dehydrogenase subunit types and Fe-S proteins were enriched only on touchscreens (Fig. 6B). Increased numbers of electron transport chain components may indicate more aerobic respiration or the presence of eukaryotic DNA (as detected by assays for chloroplasts or mitochondria). Notably, high levels were found across all KOs for the horizontal pole from the Red Line and the outdoor touchscreen from Riverside Station, although it is unlikely that these trends exclusively represented eukaryotes. Riverside station touchscreen 16S profiles included only 4.04% classified sequences of chloroplasts, and overall, the holds included for shotgun sequencing had the highest average proportions of chloroplasts, followed by chairs and touchscreens. Thus, the presence of more electron transport chain components may also reflect a metabolic strategy enriched among persisters in the built environment, especially relevant to the alphaproteobacteria detected on touchscreens.

### Minimal presence of pathogenic and antibiotic resistance on the Boston transit system.

**FIG 7:**
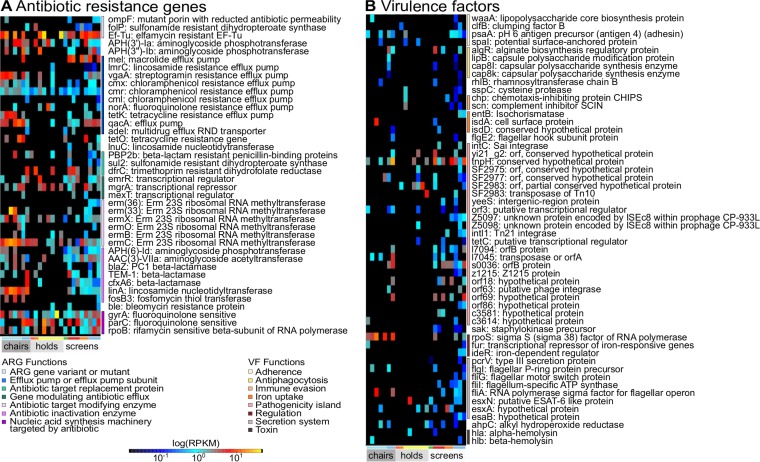
Quantification of antibiotic resistance marker and virulence factor abundances on subway surfaces. (A) Antimicrobial resistance markers (rows) quantified in metagenomes by ShortBRED (36) and annotated by antibiotic target through the use of antibiotic resistance ontology in CARD. (B) Virulence factors (rows) likewise quantified and manually annotated by virulence function through the use of keywords on the VFDB website. For both heat maps, columns (samples) are arranged as described for Fig. 6.

To detect antibiotic resistance factors in MBTA metagenomes, we used ShortBRED (Short Better Read Extract data set) (36) to create high-precision sequence markers from the Comprehensive Antibiotic Resistance Database (CARD) (37). The results included 2,657 antibiotic resistance gene (ARG) markers for 792 ARGs in CARD, but only 46 ARG markers were detected with values corresponding to the number of reads per kilobase per million reads (RPKMs) greater than 0 in at least two samples. This is notable because the average read depth of our samples was 9.8 × 10^6^ reads (0.989 gigabases) but the average RPKM per sample for these markers was only 1.172, with values ranging from 0 to 46.67. Similarly, a low abundance of ARGs (<0.3% of total reads mapped to the Antibiotic Resistance Database [ARDB]) was found in the Home Microbiome Project (2). Our hits included several resistance mechanisms, including efflux pumps, antibiotic target modification or replacement, antibiotic inactivation, and changes in nucleic acid machinery (*rpoB* or *par* genes) (Fig. 7A).

To contextualize ARG enrichment (or, rather, depletion) in this environment, we further compared the ARG enrichment in samples from the Boston subway to that of ARGs in samples of the air microbiome from several other built environments (38) as well as to those in 552 stool samples from individuals in the United States, China, Malawi, and Venezuela (12, 39, 40). For consistency with previous surveys, we used ShortBRED to generate 4,132 antibiotic ARG markers for 849 ARGs in the Antibiotic Resistance Database (ARDB). Both the air microbiome and Boston subway samples had noticeably lower levels of RPKMs than were seen with typical human stool samples (see Fig. S3 in the supplemental material). The gut microbiome has repeatedly been observed (41) to be enriched for tetracycline resistance, beta-lactamases, and MFS/RNS efflux pumps, whereas none of these were substantially present in the MBTA, and only low levels of tetracycline and beta-lactamase resistance in indoor air were determined (38).

10.1128/mSystems.00018-16.3 Figure S3  Comparison of antibiotic resistance markers from the ARDB database. Data represent RPKMs of antibiotic resistance gene markers from air microbiomes in New York City (office) and San Diego (hospital, home, and pier) and the Boston MBTA and from gut microbiomes of 552 individuals in the United States, China, Malawi, and Venezuela (12, 39, 40). Download Figure S3, TIF file, 1.6 MB. Copyright © 2016 Hsu et al. 2016 Hsu et al. This content is distributed under the terms of the Creative Commons Attribution 4.0 International license.

To similarly assess virulence factors in the MBTA, we created sequence markers from the Virulence Factor Database (VFDB) (42), which resulted in 7,869 markers for 2,089 factors. A total of 54 markers were detected with RPKMs greater than 0 in at least two samples. The average RPKM per sample was 0.240, ranging from 0 to 23.74. All of the putative virulence factors, with the exception of the alpha- and beta-hemolysin proteins found in *S. aureus*, are opportunistic factors typical of normal microbial life. For example, many proteins were classified as part of pathogenicity islands; however, most of these proteins represent transposases, integrases, and repetitive regions (Fig. 7B). Other hits were annotated with functions in adherence, antiphagocytosis, and secretion systems but consisted of cell surface proteins such as lipopolysaccharides, capsule polysaccharide proteins, and flagellar proteins. This indicates that the real pathogenic potential detected in the Boston subway is very low. Overall, the Boston subway has minimal levels of antibiotic resistance and virulence factors.

## DISCUSSION

Here, we report on the microbial profile of the Boston metropolitan transit system. Previous studies have characterized the Hong Kong and New York subway aerosol communities (7, 8), as well as surfaces in the New York subway (9), but we believe this to be the first study to have determined how space utilization by passengers, surface type, and material composition individually affect microbial ecology. We further describe the microbial community metabolic potential across surface types and metagenomically assess the absence of pathogenic potential. The former primarily reflected *P. acnes* pathways on holds and aerobic respiration on seats and touchscreens; resistance and virulence factors among the latter were depleted relative to environments such as the human microbiome.

The surface type was the major driver of variation in composition, lending support to three potential hypotheses positing that differences may be driven by (i) human body interactions (6); (ii) the material composition of these surfaces, which may enhance microbial adherence and growth; or (iii) a combination of the two factors. Our data support the third hypothesis. First, we observed a significant enrichment of oral microbes on horizontal poles and grips, which may be higher up and closer to the face of each rider or may reflect transfer through skin-mediated contact (Fig. 1C). Second, both 16S data and shotgun data showed enrichment of vaginal commensals on seat surfaces, which may be transmitted through clothing. Third, we found that seats were enriched in vaginal and oral taxa relative to seat backs and that outdoor touchscreens were enriched in alphaproteobacteria relative to indoor touchscreens. If surface material were the only driver of microbial composition, seats versus seat backs and indoor versus outdoor touchscreens should have similar taxonomic profiles. The surface material certainly plays at least a partial role, however, as we observed decreased levels of *Corynebacterium* spp. in vinyl seats compared to polyester seats. Overall, our observations indicate that both human body interactions and surface material shape community composition, with the former being the stronger driver.

Previous studies of the transit microbiome, particularly those performed in New York (9) and Hong Kong (8), have also shown environmental exposure to be an additional driver of its microbial community composition. Afshinnekoo et al., for example, found that the human DNA in samples reflected census demographics for the surrounding region (9), although we saw no differentiation at the microbial level among Boston train lines serving suburbs with different ethnodemographics. We primarily observed the impact of environmental exposure on outdoor touchscreens, in agreement with the higher alpha diversities for outdoor stations in Hong Kong reported by Leung et al. The surfaces that we investigated are nearly uniformly exposed to a high volume and diversity of rider interactions. This frequent human contact could homogenize many potential influences on microbial populations, such as demographics or weather. Since the body sites used for contact, indoor/outdoor location, and material composition remain consistent, these exposures would thus shape the taxonomic differences we observed across the Boston subway.

There are few nonopportunistic pathogens in the built environment outside hospitals (43). None were reported for restrooms (5), classrooms (6), or Hong Kong subway aerosols (8), possibly due to lack of phylogenetic resolution with 16S sequencing. During partial assembly of home (2) and rest room (44) surface metagenomes, shotgun sequencing facilitated identification of opportunists with pathogenic potential, but even with the increased resolution, outright virulence factors were rare. Robertson et al. detected no human pathogens in New York subway aerosols by the use of Sanger sequencing and pyrosequencing (7). Furthermore, although Afshinnekoo et al. reported that 12% of the taxa detected represented known pathogens in the National Select Agent Registry and PATRIC database, that database uses an extremely broad definition of “pathogen,” and these results were later refuted (10). Our study assessed whether typical subway microbial communities were unusual in their carriage or transfer of antibiotic resistance genes and virulence factors. We detected low numbers of these genes, and they were present in amounts that were drastically smaller than those observed in the human gut.

One goal of studying the microbiology of the built environment is to establish a baseline to which deviations can be compared to detect potential public health threats. As with the human microbiome, however, intersubject variability appears to be quite high in built environments (e.g., buildings) and in transit systems, and both greater cross-sectional breadth and greater longitudinal depth are still necessary. All subway microbiome papers published to date have reported a high level of skin-associated genera. In addition to this work, Leung et al. (who studied Hong Kong subway aerosols) reported results that included species of *Micrococcus* (4.9%), *Enhydrobacter* (3.1%), *Propionibacterium* (2.9%), *Staphylococcus*, and *Corynebacterium* (1.5%), while Robertson et al. detected high levels of members of the families *Staphylococcaceae*, *Moraxellaceae*, *Micrococcaceae*, *Enterobacteriaceae*, and *Corynebacteriaceae*. The report of Afshinnekoo et al. from their study of the New York subway is the only major exception, with the most abundant organisms instead found to be *Pseudomonas stutzeri*, *Acinetobacter*, and *Stenotrophomonas*. If microbes shed from skin (or still resident on shed skin cells) do dominate mass transit environments, it must be determined whether these microbes are deposited, dormant, or actively growing or whether they can be stably transferred from one individual to another.

Like those in built environments, however, human-associated microbes are by no means the only apparently functional community residents even when abundant. Notably, our samples from walls, which are not consistently touched but are in the presence of high human density, had biomass lower than and microbial compositions different from samples from other train surfaces. Establishing a “typical” microbial baseline for mass transit environments will require thoughtful sample design that controls for local space properties, short- and long-term temporal variation (e.g., time of day and season), and cross-sectional differences within and between cities. It may also prove useful to monitor for a combination of innocuous versus undesirable organisms and metabolic or functional profiles, as the results have been observed to indicate greater stability than those seen with analyses of taxonomy in the human microbiome (45). In some cases, specific pathogens may be easier to detect; in others (e.g., when individual pathogens may be extremely low density), structural, functional, or metabolic shifts may be better indicators of changing transit profiles and, consequently, of health hazards. In all such cases, future studies should incorporate expertise from architecture, engineering, public health, microbiology, and ecology, thus allowing both confident and interdisciplinary analyses as well as institutional changes in response to scientific findings.

In conjunction with other published investigations, this work helps to characterize the “urban microbiome” and, in doing so, adds to our understanding of how these microbial communities are formed, maintained, and transferred. Such studies fall in a critical space between the categories of environmental and human-associated microbial ecology and as such must address the challenges of both. Improved approaches to such studies should include designing studies with rich metadata, including architectural features, human contact, environmental exposure, surface type, and surface material; accounting for a wide range of potential biochemical environments, contaminants, and biomass levels; and involving institutional review boards, city officials, and engineers as appropriate. Future work will help to determine which urban microbes are viable and resident (as opposed to transient), as well as to identify the mechanisms utilized by the microbes to persist in the built environment. It will also be important to identify microbes that can be transferred between people via specific fomites, since this has the potential especially to inform public health and policy (by monitoring organisms or gene content or both). A greater understanding of these processes may thus eventually lead to construction of built environments that enhance and maintain human health.

## MATERIALS AND METHODS

### Study permissions.

The Massachusetts Bay Transportation Authority (MBTA) approved all aspects of our transit system sampling and gave permission to the Harvard T.H. Chan School of Public Health to conduct this study (see Fig. S4 in the supplemental material). Additional support was provided by the MBTA Police, who accompanied the study team during sample collection. A written description of the protocols and study goals was distributed to interested MBTA passengers during sampling.

10.1128/mSystems.00018-16.4 Figure S4  Letter from the MBTA. We received MBTA approval, by way of the MBTA Transit police, to carry out the study prior to grant submission and confirmed detailed sampling plans with the MBTA prior to any public work. Their assistance and input were invaluable both for study design and for safe execution of sample collection, and the letter includes the initial information from Paul MacMillan approving the work. Download Figure S4, TIF file, 1.2 MB. Copyright © 2016 Hsu et al. 2016 Hsu et al. This content is distributed under the terms of the Creative Commons Attribution 4.0 International license.

### Sample collection.

Samples were collected in 2013 on 16 May, 23 May, and 22 October from the public transit system serving metropolitan Boston during normal workday hours. Train car sampling began at the outmost termini of train routes (Alewife Station on the Red Line, Riverside Station on the Green Line, and Forest Hills Station on the Orange Line). Trains were sampled as they proceeded inbound toward the city center. Station samples were collected by swabbing the touchscreens and sides of ticket machines at five stations (Fig. 1).

For all samples, we recorded the sampling date, outdoor air temperature and relative air humidity, location, surface type (seat, seat back, horizontal pole, vertical pole, hanging grip, wall, or touchscreen), and material composition (polyester and vinyl [seats and seat backs], stainless steel [poles], polyvinyl chloride [PVC; grips], wood combinations, engineered wood, extruded thermoplastic, fiber-reinforced plastic, aluminum honeycomb panel, melamine-finished aluminum panels reinforced with Kevlar [walls], and coated glass [touchscreens]). For train car samples, we recorded the within-train location of sample collection (end or middle of car), as well as the train line and location along the route when the sample was collected. For station samples, we recorded the location of each ticketing machine (indoor, outdoor, or underground) and the side of the touchscreen swabbed (right, left, or both).

All metadata are described in Table S1 in the supplemental material, and where possible, metadata terms from the Minimum Information Standards for the Built Environment (MIxS-BE) were used (46). Weather information was compiled from weather archives from the National Oceanic and Atmospheric Administration (47) and Weather Underground (KBOS [48]).

### Swab collection and processing.

DNA-free cotton swabs (Puritan, ME, USA) were used for collection in this study. Each swab was dipped into a swabbing solution prepared from 0.15 M NaCl and 0.1% Tween 20, as used in previous studies (6, 13, 20, 49). All surfaces were swabbed for approximately 15 s, and each surface was sampled 2 or 3 times with separate swabs over nonoverlapping regions. Swabs were stored together in 15-ml Falcon tubes on ice for no more than 1 h before being taken to a central location and stored on dry ice. All samples were transported directly from dry ice to a −80°C freezer for storage.

### DNA extraction, 16S amplicon sequencing, and OTU calling.

Samples were processed using a MoBio PowerLyzer PowerSoil DNA extraction kit (Mo Bio Laboratories, Inc.). For each sample, 2 or 3 swabs from the same sample were pooled for optimal biomass recovery. Amplification and sequencing by Illumina MiSeq were performed as described previously by Caporaso et al (50). Operational taxonomic unit (OTU) tables were constructed with Quantitative Insights into Microbial Ecology (QIIME) software (51) version 1.8 (pick_closed_reference_otus.py from http://qiime.org/scripts/) with Greengenes reference version 13.5 at the 97% identity level. We filtered low-abundance OTUs (the minimum abundance threshold was 0.001 in at least 1 of 72 samples). Because the primers used in the study were designed to amplify bacterial 16S genes, we filtered out OTUs that corresponded to chloroplasts, mitochondria, and archaea. This reduced the data set to 2,134 unique OTUs representing 501 unique genera. OTU frequencies in samples were then sum-normalized to proportional data (see Table S2 in the supplemental material). Further details can be found in Text S1 in the supplemental material.

10.1128/mSystems.00018-16.10 Text S1  The supplemental information contains further detail on library construction and computational workflow, as well as results of additional analyses of contaminant taxa and a comparison to the New York subway (9). Web links to protocols and raw data are also included. Download Text S1, DOCX file, 0.04 MB. Copyright © 2016 Hsu et al. 2016 Hsu et al. This content is distributed under the terms of the Creative Commons Attribution 4.0 International license.

### Analysis methods.

Alpha diversity was calculated using the inverse Simpson diversity index in the R package “vegan” (52). Ordinations were calculated by principal coordinate analysis (PCoA) using Bray-Curtis dissimilarity, unless otherwise noted, and the relative abundance table generated above. For univariate and multivariate tests, we further filtered OTUs (the minimum abundance threshold was 0.001 in at least 7 of 72 samples). A univariate test for taxa differentially abundant with respect to touchscreen location was performed using LEfSe (11). For this analysis, each metadata category was tested using an alpha value of 0.05 for both the Kruskal-Wallis and Wilcoxon tests with one-against-all comparison and a linear discriminant analysis (LDA) effect size cutoff of 2.0. Significant univariate associations of taxa and metadata are listed in Table S3 in the supplemental material. Multivariate association tests for taxa that were differentially abundant with respect to metadata were performed using MaAsLin (27). For this analysis, we used four metadata categories: locale (train or station), surface type (e.g., seat, seat back, etc.), surface material (e.g., polyvinyl chloride, carpet, etc.), and location (e.g., Forest Hills Station, Orange Line train, etc.). Microbial source prediction was performed using Microbial Sourcetracker (22) and data from human and environmental sites reported by Hewitt et al (23). GraPhlAn (53) was used for visualization of associations and phylogenetic relationships.

### Shotgun library sequencing and quality control.

DNA was extracted using a MoBio PowerLyzer PowerSoil DNA extraction kit (Mo Bio Laboratories, Inc.) as described for 16S sequencing libraries. Only samples consisting of at least 80 ng/µl were selected and sent to the Broad Institute for shotgun library construction. Libraries were constructed using the Illumina Nextera XT method and sequenced on an Illumina HiSeq 2000 platform with 100-bp paired-end (PE) reads. The sequencing depth was 16.7 × 10^6^ PE reads per sample. The KneadDATA v0.3 pipeline (http://huttenhower.sph.harvard.edu/kneaddata) was used to remove low-quality reads and human host sequences. Further details can be found in Text S1 in the supplemental material.

### Taxonomic and functional profiling of metagenomes.

Pan-microbial (bacterial, archaeal, viral, and eukaryotic) taxonomy was determined using MetaPhlAn2 (54) (http://huttenhower.sph.harvard.edu/metaphlan2). A total of 1,340 microbial clades comprising 499 species were identified (see Table S2 in the supplemental material) and filtered for relative abundance of ≥0.1% in at least two samples for downstream multivariate analysis performed with MaAsLin (27). For all MaAsLin analyses involving shotgun taxonomic and functional profiles, we used one metadata category, namely, collapsed surface types, which included chairs (seat and seat backs), holds (grips and horizontal and vertical poles), and touchscreens.

Functional genomic profiles were generated with HUMAnN2 version 0.3.0 (55) (http://huttenhower.sph.harvard.edu/humann2), which leverages the UniRef (29) orthologous gene family catalog, along with the MetaCyc (56), UniPathway (57), and KEGG (58) databases. HUMAnN2 gives three outputs: the (i) UniRef proteins and their abundances in reads per kilobase (RPK); (ii) the MetaCyc pathways and their abundances in RPK; and (iii) the MetaCyc pathways and their coverage, ranging from 0 to 1. HUMAnN2 further calculates the RPK and coverage for each microbial taxon observed in MetaPhlAn2 for each UniRef protein and MetaCyc pathway.

To look at the functional profile, we collapsed 3,975,869 UniRef50 protein families into 12,074 Kegg Orthology (KO) numbers. UniRef50 proteins that did not belong to any KOs were not analyzed further. We sum-normalized KO RPKs and focused on KOs with mean abundance greater than the overall median abundance and variances in the 90th percentile. We identified KOs that were significantly enriched on chairs, holds, and touchscreens using MaAsLin (27) with a false-discovery rate (FDR) of <0.05. KO differences between surface types were heavily influenced by the presence of *P. acnes*. To remove this influence, we removed *P. acnes*’ RPK contribution to each UniRef50 protein and then resummed the overall UniRef50 RPK from the remaining taxa. UniRef proteins were again collapsed into KOs and subjected to the analysis described above. We then compared KOs that were significantly enriched on seats, holds, and touchscreens before and after *P. acnes* removal. Tables with KO RPKs are available at http://huttenhower.sph.harvard.edu/MBTA2015.

### Identification and quantification of antibiotic resistance and virulence factor gene markers.

Antibiotic resistance gene markers were generated with ShortBRED (36) from the Comprehensive Antibiotic Resistance Database (CARD) (37) using UniRef90 (59) as a reference. ShortBRED virulence factor markers were generated from the Virulence Factor Database (VFDB) (42) using UniRef50 (59) as a reference (due to the availability of a previous version of these markers). ShortBRED maps the shotgun reads against the markers and returns normalized marker abundances as reads per kilobase per million reads (RPKM). We aggregated and annotated antibiotic resistance gene markers using the antibiotic resistance ontology (ARO) numbers in CARD.

To facilitate cross-data set comparison, we also generated 121-bp markers with ShortBRED from the Antibiotic Resistance Database (ARDB) (60) using UniRef50 (59) as a reference and aggregated these markers at the ARDB family level. We compared the distribution of antibiotic resistance gene markers in our data set to the distributions in four previously profiled shotgun datasets corresponding to the gut microbiomes of 552 individuals in the United States (12, 40), China (39), Malawi (40), and Venezuela (40), as well as to the distribution in one shotgun data set profiling air microbiomes in a home, a hospital (indoor and outdoor), a pier, and offices (indoor and outdoor) (38). Virulence factors were annotated using VFDB ontologies available at http://www.mgc.ac.cn/VFs/main.htm. ShortBRED results can be found in Table S5 in the supplemental material.

10.1128/mSystems.00018-16.9 Table S5  Antibiotic resistance gene and virulence factor markers. Data represent RPKM values for CARD (first tab), VFDB (second tab), and ARDB (third tab). The RPKM values for CARD and VFDB represent MBTA data only; the ARDB data represent values from multiple shotgun datasets (12, 38–40). Download Table S5, XLSX file, 1.6 MB. Copyright © 2016 Hsu et al. 2016 Hsu et al. This content is distributed under the terms of the Creative Commons Attribution 4.0 International license.

### Nucleotide sequence accession number.

Raw sequence files were deposited into the Sequence Read Archive (SRA) of the National Center for Biotechnology Information (NCBI) with accession no. PRJNA301589.
