# Supplementary figures and images for: Urban Transit System Microbial Communities Differ by Surface Type and Interaction with Humans and the Environment
Source: mSystems. 2016 Jun 28;1(3):e00018-16. doi: 10.1128/mSystems.00018-16 (PMC5069760; doi:10.1128/mSystems.00018-16)

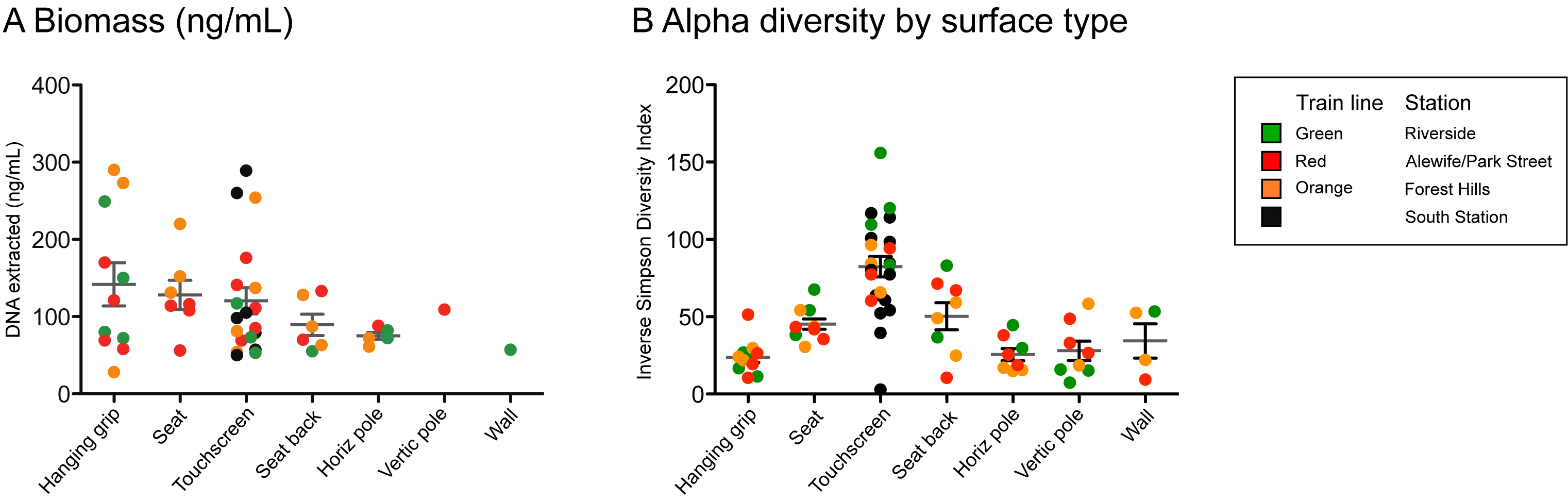

Supplement: Figure S1 [file sys003162033sf2.tif]

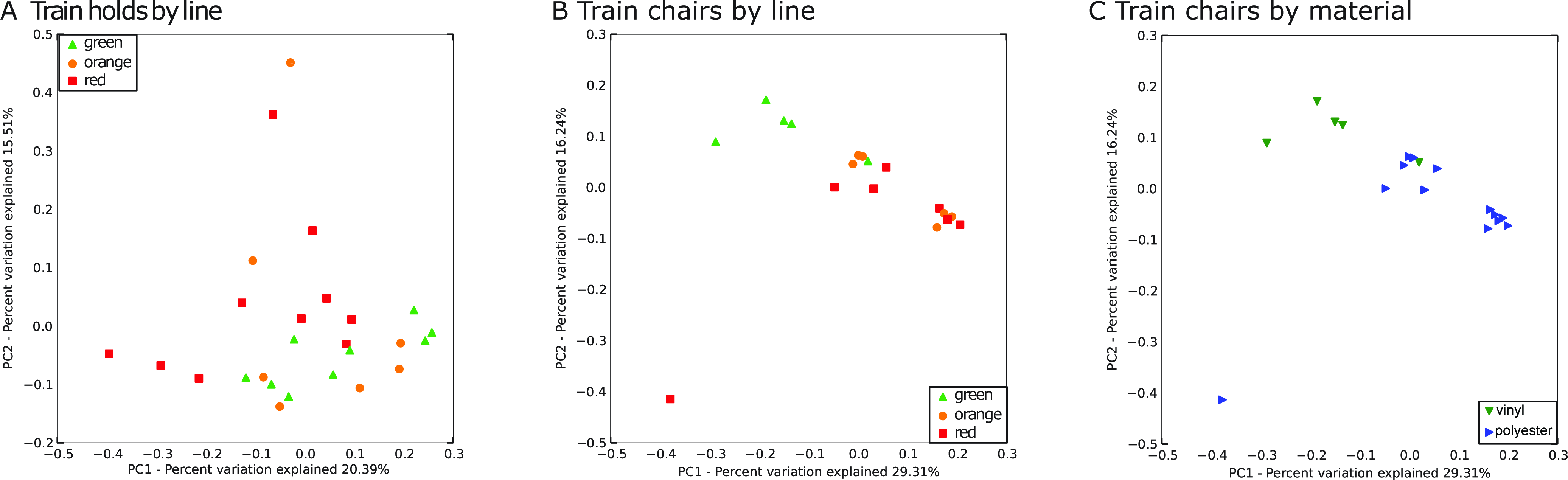

Supplement: Figure S2 [file sys003162033sf3.tif]

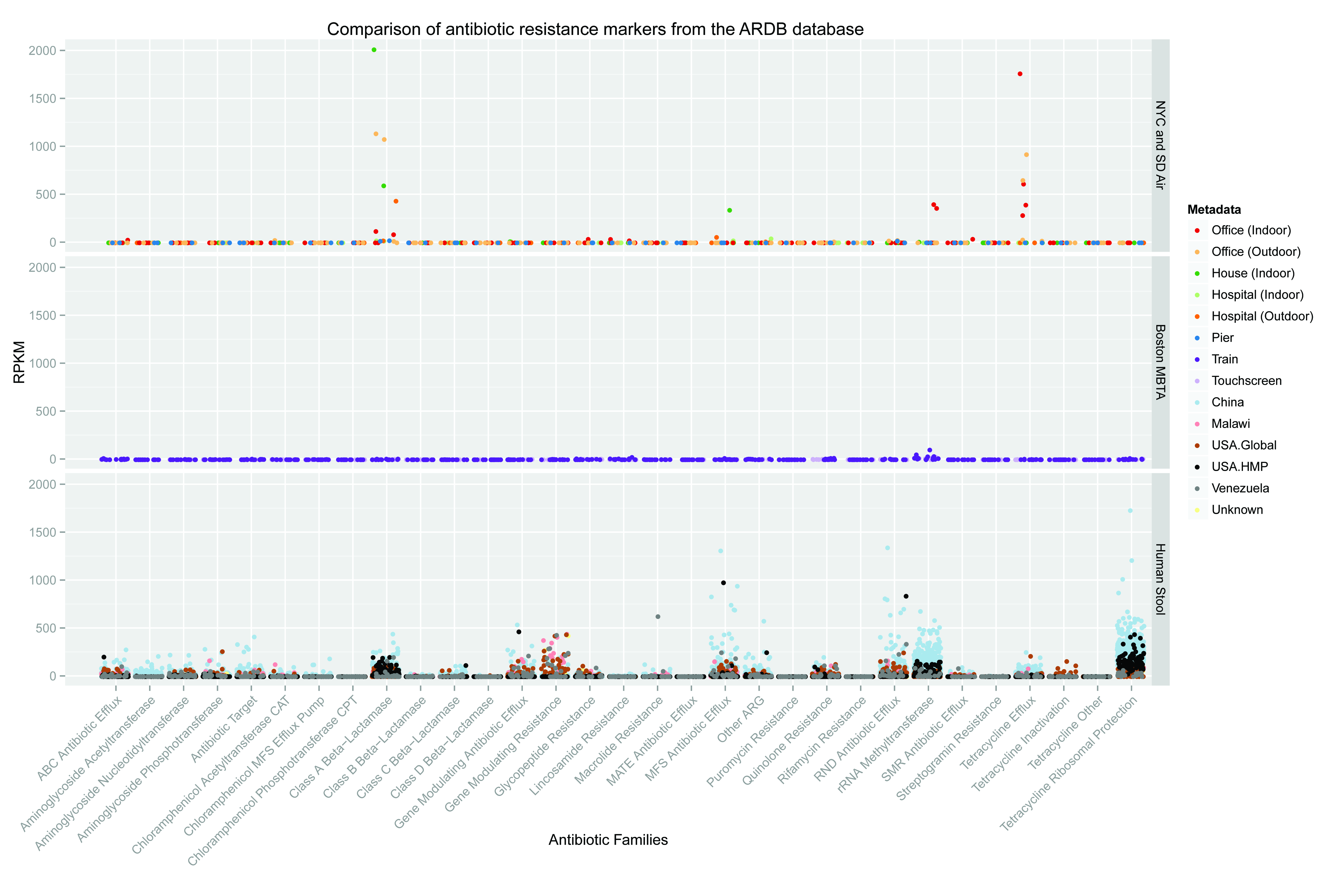

Supplement: Figure S3 [file sys003162033sf4.tif]

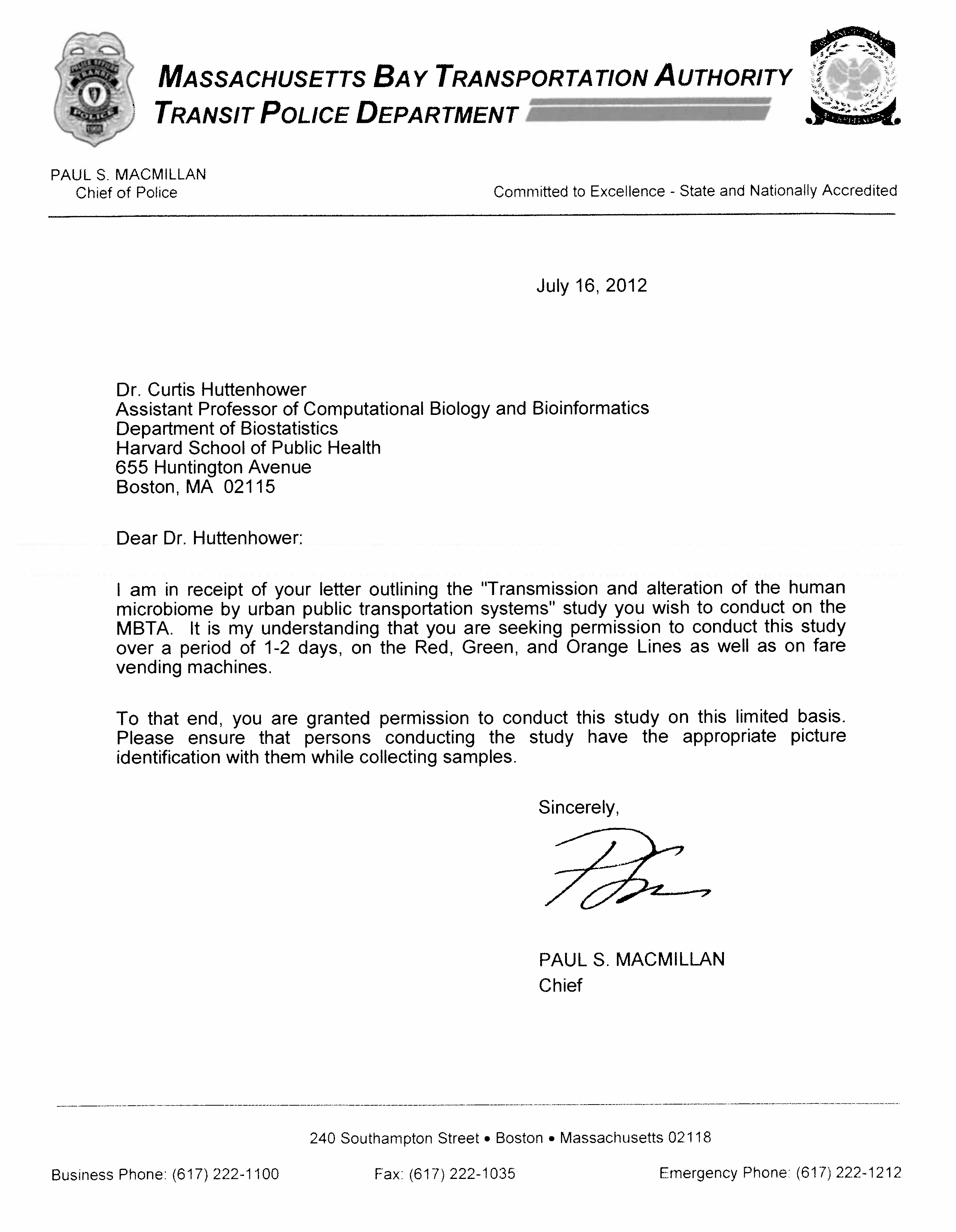

Supplement: Figure S4 [file sys003162033sf5.tif]
