# Supplementary material for: Urban Transit System Microbial Communities Differ by Surface Type and Interaction with Humans and the Environment
Source: mSystems. 2016 Jun 28;1(3):e00018-16. doi: 10.1128/mSystems.00018-16 (PMC5069760; doi:10.1128/mSystems.00018-16)
Supplement: Text S1 [file sys003162033s1.docx]

Supplemental Information

The BioProject number, protocols, raw data tables, and supplemental tables can be downloaded at <http://huttenhower.sph.harvard.edu/MBTA2015>.

## Methods and Materials

**DNA extraction, 16S amplification and sequencing.** Samples were processed using the MoBio PowerLyzer PowerSoil DNA extraction kit (MO BIO Laboratories, Inc.) using bead-beating homogenization. For each sample, 2 or 3 swabs from the same sample were pooled for optimal biomass recovery.  Each swab was individually homogenized in a bead-beating tube at 6.0 M/s for 40 seconds on the MP Biomedical FastPrep 24, but subsequent cleanup was pooled over one column. Extracted DNA extracts were quantified using a Qubit fluorimeter and sent to the Broad Institute for sequencing. Amplification and sequencing by Illumina MiSeq were performed as described previously (1). In brief, genomic DNA was subjected to 16S amplification using primers designed incorporating the Illumina adapters and a sample barcode sequence, allowing directional sequencing covering variable region V4 (Primers: 515F[GTGCCAGCMGCCGCGGTAA] and 806R [GGACTACHVGGGTWTCTAAT]). PCR was performed in triplicate with 1 μl of template (1:50), 10 μl of HotMasterMix with the HotMaster Taq DNA Polymerase (5 Prime), and 1 μl of primer mix (for final concentration of 10 μM). The cycling conditions consisted of an initial denaturation of 94°C for 3 min, followed by 24 cycles of denaturation at 94°C for 45 sec, annealing at 50 °C for 60 sec, extension at 72°C for 5 min, and a final extension at 72°C for 10 min. Amplicons were quantified on the Caliper LabChipGX (PerkinElmer, Waltham, MA), pooled in equimolar concentrations, size selected (375-425 bp) on the Pippin Prep (Sage Sciences, Beverly, MA) to reduce non-specific amplification products from host DNA, and a final library size and quantification was performed on an Agilent Bioanalyzer 2100 DNA 1000 chip (Agilent Technologies, Santa Clara, CA). Sequencing was performed on the Illumina MiSeq platform (version 2) according to the manufacturer’s specifications with addition of 5% PhiX, and yielded paired-end reads of 150 bp in length in each direction. Total read depth was at least 5,000 reads (up to over 100,000 reads) per sample.

**OTU calling.** Quantitative Insights into Microbial Ecology (QIIME) software (2) version 1.8 was used for data processing. Paired-end reads (with approximately 97 bp overlap) were stitched and size selected (225 – 275 bp) to reduce nonspecific amplification products. Operational taxonomic units (OTUs) were called with a closed reference (pick_closed_reference_otus.py) using the Greengenes reference version 13.5 at the 97% identity level based on the PICRUSt(3) protocol.  Using these parameters, we observed 17,954 unique OTUs. We filtered low-abundance OTUs (minimum abundance threshold 0.001 in at least one of 72 samples); this reduced the dataset to 2,134 unique OTUs representing 501 unique genera. Since the primers used in the study were designed to amplify bacterial 16S genes, we filtered out OTUs that corresponded to chloroplasts, mitochondria, and archaea. OTU frequencies in samples were then sum-normalized to proportional data. The filtered OTU tables can be found in **Table S2**.

**KneadData**. KneadData incorporates Trimmomatic(4) and bowtie2(5) for filtering and human sequence removal, respectively. Reads were scanned with a four-base wide sliding window and trimmed when the average base Phred score drops below 20. Trimmed reads shorter than 70 nt were discarded. UCSC Human genome assembly version hg38 was used as reference for removal of human sequences. The average sequencing depth after quality control was 9.8×10^6^ reads per sample.

**Negative control analyses.** Unfortunately, our study did not include negative controls beyond those internal to the sequencing platform. Instead, we took several measures during analysis to test for contamination in the 16S datasets. First, we looked at relative abundances across multiple sets of samples on the same sequencing plate, since taxa present across all samples may indicate contamination (especially since the batch included many non-transit samples). This was possible mainly for the touchscreen samples (n=21) and a few train samples (n=6), which were pooled with 30 saliva cultures, 13 skin samples, and 2 macaque adipose samples. At the species level, we found 42 taxa (of 1647 total) that were present in 80% of samples, with average abundance ranging from 0.018% (*Pseudomonas* unknown) to 11.1% (*Actinomyces* unknown). Many of these are skin-associated, including *Pseudomonas, Staphylococcus, Corynebacterium* (in increasing abundance) or associated with the oral cavity, including *Fusobacterium, Veillonella, Peptostreptococcus, Streptococcus, Prevotella,* and *Porphyromonas* (in increasing order) (**Table S2**). It is unclear whether the latter arises from the large number of saliva samples in this dataset, or as a true contaminant. None of the taxa with lower average abundance are key to our findings.

Chloroplast and mitochondrial sequences were actually considered to be a type of contaminant in our study, inasmuch as they essentially represent plant- and human-material derived reads. They were found across all touchscreen and surface samples, but at very low levels in adipose fat (primate, not human-derived) and saliva. Others have claimed that chloroplast DNA may be an artifact of cotton swabs rather than environmental exposures; our skin samples were processed with Copan swabs and yielded 1-2 orders of magnitude fewer chloroplast sequences (<1% maximum). Our standard primer pairs are known to amplify chloroplast and mitochondrial sequences: this is a well-known problem for those that study plant-associated microbial communities (6, 7). Chloroplast DNA percentages varied from 1.32%-6.98%, and 0.054-1.03% in the touchscreens. They varied even more in the train data (not pooled with the touchscreens): chloroplast DNA ranged from 0.9% to 62.39%, with especially high levels on the Red line, while mitochondrial DNA varied from 0-8.27% on the trains (data available via website). This led to our analysis strategy of treating both sequence types like typical contaminants, discounting their sequence abundance, renormalizing, and analyzing primarily the resulting quality-controlled datasets.

Physical negative controls should be part of future study design, as recommended by Adams et al (8) and Salter et al (9). Their use, we note, must still be context dependent, as no one blanket analysis is likely to apply to different sample and contaminant types. Some studies have utilized the approach outlined in Flores et al, where OTUs constituting greater than 1% of the total negative control sequences were removed from all samples prior to rarefaction and analyses (10). Another approach developed by Meadow et al involves searching for taxa with high abundance in negative controls relative to samples: this is done by plotting the relative abundances of taxa in negative controls against the relative abundances of taxa in samples and applying a cutoff (11). Adams et al performed a meta-analysis of built environment studies, and reported phylum Tenericutes as significantly enriched in kit microbiomes, and Cyanobacteria (or chloroplast) as highly abundant in dust but not in kits. They also mention that skin taxa are often found as contaminants, but removing them could remove true signal. Typical kit contaminant taxa were also not significant in our study.

## Comparison to the NY subway study

To expand our comparison with the previous NYC subway study, we downloaded their MetaPhlAn2 tables (provided at the time of the NYC publication by Nicola Segata in collaboration with our group) from their supplementary data. We applied a simple quality control filter by retaining taxa with at least 0.1% abundance in at least 1% of samples (14 samples), and then focused specifically on the samples most similar to ours, i.e. from subway stations or trains.

In the NYC study, the most abundant taxa in the resulting 1,416 samples included *Pseudomonas stuzeri* (27.01%), *Pseudomonas unclassified* (8.66%), *Enterobacter cloacae* (7.66%)*, Stenotrophomonas maltophilia* (7.10%)*,* and *Acinetobacter pitti/calcoaceticus/nosocomialis* (3.39%). Neither *Yersinia* nor *Bacillus anthracis* were present in any samples. These results are strikingly different from our top species from similarly analyzed metagenomic data, which included *Propionibacterium acnes* (47.44%), *Propionibacterium* phage (total ~6%), *Micrococcus luteus* (2.40%)*,* and *Staphylococcus epidermis* (1.98%). This may be due to a combination of factors, most likely the different types of surfaces sampled, but also including the swab protocol development and biomass validation prior to sequencing carried out for our study (see Methods). Most of our samples represent heavily utilized, nonporous, non-sanitized surfaces within train cars or, less often, stations; in contrast, NYC study surfaces include benches (n=326), rails/poles (condensed from other categories, n=468), garbage cans (n=142), kiosks (n=161), turnstiles (n=151), and doors (n=77), with all other surfaces sampled <24 times.

In support of this hypothesis, the NYC microbiomes at least in part do resemble those of other built environment surfaces and dust. Adams et al, for example (12), collected dust in vacuums or passively (through settlement). The former, which was considered homogenized, had significantly higher levels of Pseudomonales, Enterobacteriales, and Streptophyta as compared to the latter. Overall, Gammaproteobacteria dominated most samples (ave. 76.8%), still primarily from Pseudomonadales and Enterobacteriales, and overshadowed the Bacilli (6.68%), Betaproteobacteria (5.02%), and Alphaproteobacteria (4.80%), and Actinobacteria (2.48%). The NYC subway had high levels of Enterobacteriales (17.90%) and Pseudomonadales (49.61%), but none for Streptophyta (0%, suggesting a possible sampling or extraction bias). However, it is difficult to compare NYC swabbed samples (or our own) to vacuumed or settled dust, given the extreme heterogeneity seen in the latter for distinct space types or time integration periods. Adams et al, for example, was in turn quite distinct from dust in the International Space Station (13), for example, a mixed use academic classroom building (14), or house dust (15), none of which significantly resembling our skin-dominated MBTA surfaces.

Taking these unusual features of the NYC subway data as given, however, we sought to determine whether surface material was at least a major determinant of their microbial community composition, as it proved to be for ours. We grouped their sample metadata into four categories: type of object (bench, rail/pole, garbage can, kiosk, turnstiles, etc.), surface material (wood, metal, plastic, etc.), object category (station, train, etc.), and borough (Queens, Brooklyn, Manhattan, etc.) Applying the MaAsLin multivariate linear model to these variables jointly, we found 71 differentially abundant clades at FDR<0.25.

Surprisingly, none of these associations were with surface material type; most instead segregated with object type, which may at least be concordant with the much greater diversity of objects sampled in the NYC study. Rails and poles had lower levels of *Pseudomonas* and *Acinetobacter lwoffi* as compared to benches, for example, while garbage cans had higher levels of *Enterococcus italicus* and *Leunostoc*. Clostridia and *Klebsiella* (not marine taxa) were found in the abandoned South Ferry and Penn Station timecourse samples, as well as in trains as compared to all other stations. Lastly, and also surprising, some taxa were associated with borough: this includes higher levels of Acinetobacter and Moraxellaceae in Manhattan as compared to the Bronx. Without more detail on the study’s exact sampling protocol - which parts of these diverse objects were swabbed, for example, and for how long over what surface area - it is difficult to interpret statistically significant but low effect size differences. It may be useful for future studies to sample fewer, more controlled environments with greater specificity, and of course to assess the results with more careful and targeted metagenomic analyses.

References

1. **Caporaso JG, Lauber CL, Walters WA, Berg-Lyons D, Lozupone CA, Turnbaugh PJ, Fierer N, Knight R.** 2011. Global patterns of 16S rRNA diversity at a depth of millions of sequences per sample. Proc Natl Acad Sci U S A **108 Suppl 1:**4516-4522.

2. **Caporaso JG, Kuczynski J, Stombaugh J, Bittinger K, Bushman FD, Costello EK, Fierer N, Pena AG, Goodrich JK, Gordon JI, Huttley GA, Kelley ST, Knights D, Koenig JE, Ley RE, Lozupone CA, McDonald D, Muegge BD, Pirrung M, Reeder J, Sevinsky JR, Turnbaugh PJ, Walters WA, Widmann J, Yatsunenko T, Zaneveld J, Knight R.** 2010. QIIME allows analysis of high-throughput community sequencing data. Nature methods **7:**335-336.

3. **Langille MG, Zaneveld J, Caporaso JG, McDonald D, Knights D, Reyes JA, Clemente JC, Burkepile DE, Vega Thurber RL, Knight R, Beiko RG, Huttenhower C.** 2013. Predictive functional profiling of microbial communities using 16S rRNA marker gene sequences. Nat Biotechnol **31:**814-821.

4. **Bolger AM, Lohse M, Usadel B.** 2014. Trimmomatic: a flexible trimmer for Illumina sequence data. Bioinformatics **30:**2114-2120.

5. **Langmead B, Salzberg SL.** 2012. Fast gapped-read alignment with Bowtie 2. Nat Methods **9:**357-359.

6. **Rastogi G, Tech JJ, Coaker GL, Leveau JH.** 2010. A PCR-based toolbox for the culture-independent quantification of total bacterial abundances in plant environments. J Microbiol Methods **83:**127-132.

7. **Lane D.** 1991. 16S/23S rRNA sequencing, p 115-175. *In* Stackebrandt E GM (ed), Nucleic acid techniques in bacterial systematics. John Wiley and Sons, Chichester, United Kingdom.

8. **Adams RI, Bateman AC, Bik HM, Meadow JF.** 2015. Microbiota of the indoor environment: a meta-analysis. Microbiome **3:**49.

9. **Salter SJ, Cox MJ, Turek EM, Calus ST, Cookson WO, Moffatt MF, Turner P, Parkhill J, Loman NJ, Walker AW.** 2014. Reagent and laboratory contamination can critically impact sequence-based microbiome analyses. BMC Biol **12:**87.

10. **Flores GE, Henley JB, Fierer N.** 2012. A direct PCR approach to accelerate analyses of human-associated microbial communities. PLoS One **7:**e44563.

11. **Meadow JF, Altrichter AE, Green JL.** 2014. Mobile phones carry the personal microbiome of their owners. PeerJ **2:**e447.

12. **Adams RI, Tian Y, Taylor JW, Bruns TD, Hyvarinen A, Taubel M.** 2015. Passive dust collectors for assessing airborne microbial material. Microbiome **3:**46.

13. **Checinska A, Probst AJ, Vaishampayan P, White JR, Kumar D, Stepanov VG, Fox GE, Nilsson HR, Pierson DL, Perry J, Venkateswaran K.** 2015. Microbiomes of the dust particles collected from the International Space Station and Spacecraft Assembly Facilities. Microbiome **3:**50.

14. **Kembel SW, Meadow JF, O'Connor TK, Mhuireach G, Northcutt D, Kline J, Moriyama M, Brown GZ, Bohannan BJ, Green JL.** 2014. Architectural design drives the biogeography of indoor bacterial communities. PLoS One **9:**e87093.

15. **Lax S, Smith DP, Hampton-Marcell J, Owens SM, Handley KM, Scott NM, Gibbons SM, Larsen P, Shogan BD, Weiss S, Metcalf JL, Ursell LK, Vazquez-Baeza Y, Van Treuren W, Hasan NA, Gibson MK, Colwell R, Dantas G, Knight R, Gilbert JA.** 2014. Longitudinal analysis of microbial interaction between humans and the indoor environment. Science **345:**1048-1052.
